# A Survey on Deep Learning-Based Short/Zero-Calibration Approaches for EEG-Based Brain–Computer Interfaces

**DOI:** 10.3389/fnhum.2021.643386

**Published:** 2021-05-28

**Authors:** Wonjun Ko, Eunjin Jeon, Seungwoo Jeong, Jaeun Phyo, Heung-Il Suk

**Affiliations:** ^1^Department of Brain and Cognitive Engineering, Korea University, Seoul, South Korea; ^2^Department of Artificial Intelligence, Korea University, Seoul, South Korea

**Keywords:** brain–computer interface, electroencephalography, deep learning, data augmentation, transfer learning

## Abstract

Brain–computer interfaces (BCIs) utilizing machine learning techniques are an emerging technology that enables a communication pathway between a user and an external system, such as a computer. Owing to its practicality, electroencephalography (EEG) is one of the most widely used measurements for BCI. However, EEG has complex patterns and EEG-based BCIs mostly involve a cost/time-consuming calibration phase; thus, acquiring sufficient EEG data is rarely possible. Recently, deep learning (DL) has had a theoretical/practical impact on BCI research because of its use in learning representations of complex patterns inherent in EEG. Moreover, algorithmic advances in DL facilitate short/zero-calibration in BCI, thereby suppressing the data acquisition phase. Those advancements include data augmentation (DA), increasing the number of training samples without acquiring additional data, and transfer learning (TL), taking advantage of representative knowledge obtained from one dataset to address the so-called data insufficiency problem in other datasets. In this study, we review DL-based short/zero-calibration methods for BCI. Further, we elaborate methodological/algorithmic trends, highlight intriguing approaches in the literature, and discuss directions for further research. In particular, we search for *generative model*-based and *geometric manipulation*-based DA methods. Additionally, we categorize TL techniques in DL-based BCIs into *explicit* and *implicit* methods. Our systematization reveals advances in the DA and TL methods. Among the studies reviewed herein, ~45% of DA studies used generative model-based techniques, whereas ~45% of TL studies used explicit knowledge transferring strategy. Moreover, based on our literature review, we recommend an appropriate DA strategy for DL-based BCIs and discuss trends of TLs used in DL-based BCIs.

## 1. Introduction

### 1.1. Overview

Brain–computer interfaces (BCIs) (Dornhege et al., 2007; Lotte et al., 2018; Roy et al., 2019) provide communication pathways between a user and an external device (e.g., robotic arm, speller, seizure alarm system, etc.) by measuring and analyzing brain signals. Owing to its practicality, non-invasive BCIs based on electroencephalography (EEG) are commonly exploited (Suk and Lee, 2012; Roy et al., 2019). The *real-world* impact of BCIs is promising because they can identify intention-reflected brain activities. In the past decade, human-centered BCIs, such as those in mental fatigue detection tasks (Binias et al., 2020; Ko et al., 2020b), emotion recognition (Qing et al., 2019), and controlling exoskeletons (Lee et al., 2017) have shed light on the success of improving human ability. An *active* BCI (Fahimi et al., 2020) recognizes complex patterns from EEG spontaneously caused by a user's intention independent of external stimuli, and a *reactive* BCI (Won et al., 2019) identifies brain activities in reaction to external events. A *Passive* BCI (Ko et al., 2020b) is exploited to acquire implicit information of a user's cognitive status without any voluntary control.

EEG-based BCIs generally benefit from machine learning techniques (Lotte et al., 2018). Specifically, EEG features of various paradigms are crafted using machine learning algorithms, such as *common spatial pattern* (CSP) (Ramoser et al., 2000) and *canonical correlation analysis* (Lin et al., 2006), including preprocessing techniques. Further, the extracted EEG features are discriminated by successful machine learning algorithms used in classification tasks, e.g., *support vector machines* (Bishop, 2006). These feature extraction and classification algorithms have shown their ability in EEG-based BCIs but have also been limited because of the lack of representation power for complex EEG patterns (Schirrmeister et al., 2017). In addition, since feature extractions using these machine learning methods are widely performed in a *hand-crafted manner* (Lawhern et al., 2018), it is difficult for *unskilled personnel* to develop a novel BCI framework.

Deep learning (DL) methodologies (Schirrmeister et al., 2017; Sakhavi et al., 2018; Zhang et al., 2019c; Ko et al., 2020a) have become the core of BCI research owing to their representational power for complex patterns in EEG. Specifically, DL significantly simplifies the EEG analysis pipeline (Lawhern et al., 2018) by learning preprocessing, feature representation, and decision-making in an *end-to-end* manner. Furthermore, architectural developments in DL have been very successful in representing complicated patterns. DL learns the hierarchical representations of input data through stacked non-linear transformations (LeCun et al., 2015). In DL, stacked layers apply a linear transformation to the input, and the transformation is fed through non-linear activation. The parameters of these stacked layers are automatically learned by exploiting an *objective* function. In the machine learning field, various DL architectures have been developed. Examples include convolutional neural networks (CNNs), which have been well-suited for *structural* pattern representation and are thus widely used to learn *spatio-spectral-temporal* patterns of EEG (Schirrmeister et al., 2017; Ko et al., 2020a). Additionally, owing to the ability of sequential data modeling, recurrent neural networks and their variants, e.g., long short-term memory (LSTM) networks, have achieved considerable success in the temporal embedding of EEG (Zhang et al., 2019c; Freer and Yang, 2020). Moreover, recent research has shown interest in hybrid forms of recurrent layers and convolutional layers (Ko et al., 2018; Zhang et al., 2019a).

Although DL has been demonstrated to be a powerful tool in EEG analysis, there are some limitations. First, typically available EEG datasets contain substantially fewer training samples than do other datasets that are commonly used in DL-based *computer vision* or *natural language processing* task development. However, EEG acquisition is an expensive and time-consuming task. Further, data accessibility is often hindered because of privacy concerns, especially in the clinical domain. Thus, collecting large amounts of training EEG samples for DL training is rarely possible. Owing to the nature of EEG properties, such as low signal-to-noise ratio and inter/intra-variability (Jayaram et al., 2016), DL-based BCIs are rarely trained only with a different user's or even multiple users' training EEG samples.

To address the aforementioned problems, recent research has focused on *data augmentation* (DA) (Luo and Lu, 2018; Zhang et al., 2019d; Fahimi et al., 2020) and *transfer learning* (TL) (Jayaram et al., 2016; Kwon et al., 2019; Jeon et al., 2020). The use of DL has shown the possibility of synthesizing high-dimensional image data (Goodfellow et al., 2014), audio data (Donahue et al., 2019), and EEG data (Hartmann et al., 2018). Further, traditional DA techniques used in DL fields, such as image rotation have demonstrated their own efficiency and effectiveness (Simonyan and Zisserman, 2014). By exploiting these DA techniques, DL-based BCIs have improved the performance with a short-calibration phase producing little data (Fahimi et al., 2020; Zhang et al., 2020b). In terms of TL, DL has also been widely used to suppress the training EEG data acquisition phase (Chai et al., 2016; Jeon et al., 2020; Tang and Zhang, 2020). In particular, DL-based BCIs can be designed in a short/zero-calibration manner by appropriately conducting 2-fold TL strategies, i.e., explicit TL and implicit TL.

Overall, several DL methods have been proven to improve existing EEG processing techniques. The end-to-end strategy allows DL to simply learn existing EEG analysis pipelines, reducing paradigm-specific processing and feature extraction. Objective function-based automatic learning requires only raw or minimally preprocessed EEG data. The feature representation of DL can also be more effective and richer than features engineered by humans. Moreover, DL can pave the way for methodological advances in EEG analysis, such as generative modeling (Goodfellow et al., 2014) and knowledge transfer (Jayaram et al., 2016) to handle the lack of EEG data problems and the data variability issue.

### 1.2. Our Contributions

**Figure 1:**
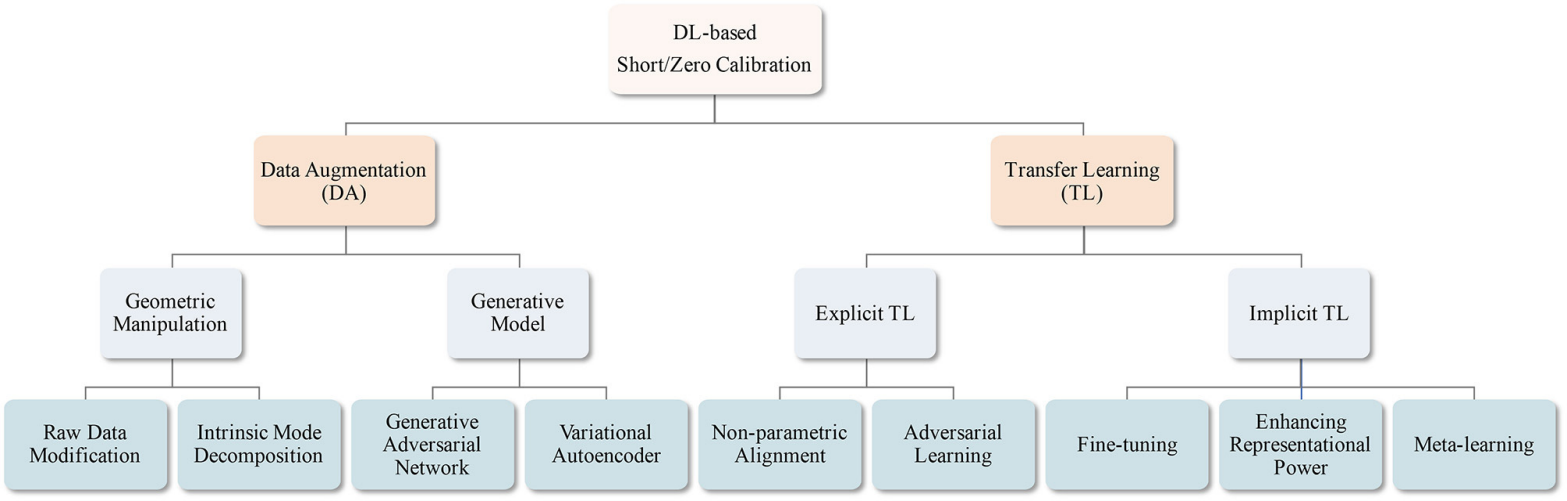
Overview of DL-based short/zero calibration approaches.

In this study, we review DL-based BCI studies that mostly focused on suppressing the EEG calibration phase. Unlike recent survey papers for EEG-based BCIs that are mostly focused on introducing machine learning/DL algorithms for BCIs (Lotte et al., 2018; Craik et al., 2019; Zhang et al., 2020d), summarizing EEG analysis studies (Roy et al., 2019), providing comprehensive information on EEG-based BCIs, including sensing technology and healthcare systems (Gu et al., 2020), and surveying application of machine learning/DL-based TLs (Zhang et al., 2020c), our review aims to address short-/zero-calibration techniques for EEG-based BCIs. In detail, we categorize these studies into two different groups, based on the manner of increasing the number of training samples: (i) manipulating the given training data without using an additional one and (ii) exploiting other subjects/sessions' EEG samples. Specifically, (i) is further categorized into generative model-based and geometric manipulation-based methods, and (ii) is classified into explicit and implicit knowledge transfer. In the case of (i), 45% of the studies proposed generative model (Goodfellow et al., 2014; Kingma and Welling, 2014)-based DA methodologies, whereas 45% of the case of (ii) developed explicit knowledge transfer strategies. Further, we recommend a training technique for DL-based BCI models with a generative model-based DA based on our literature review and discuss trends of recent knowledge transfer methods. We summarize the taxonomy of our review in Figure 1.

The remainder of this paper is organized as follows. In section 2, we describe DL methods to augment training samples and review the methods proposed in various BCI studies. In section 3, we discuss and review DL methods for transferring knowledge of other subjects/sessions' samples in BCIs. For both sections 2 and 3, we summarize our review in Tables 1–4. Section 4 presents our discussion and recommendations for DA-based short-calibration techniques to develop a new DL-based BCI system. Further, section 4 details trends of recent knowledge transfer methods in DL research. Finally, section 5 provides concluding statements.

**Table 1:** Geometric manipulation data augmentation methods.

	**Approach**	**References**	**Paradigm**	**Summary**
Raw data modification	Geometric transformation	Zhang et al., 2020b	Motor imagery	Rotated (180°), shifted, and changed RGB values of STFT images estimated from raw EEGs
		Shovon et al., 2019		Rotated (5°), flipped, zoomed, brightened (±30%) STFT images estimated from raw EEGs
		Schirrmeister et al., 2017		Cropped raw EEG using a sliding window
		Ko et al., 2018		Cropped raw EEG using a sliding window
		Majidov and Whangbo, 2019		Cropped raw EEG using a sliding window
		Freer and Yang, 2020		Flipped raw EEG
		Mousavi et al., 2019	Sleep	Cropped raw EEG using a sliding window
		Supratak and Guo, 2020		Shifted raw EEG
		Sakai et al., 2017	Cognition	Shifted raw EEG
	Noise addition	Zhang et al., 2020b	Motor imagery	Added Gaussian noise (std of 0.1)
		Freer and Yang, 2020		Used uniform noise ([−0.5, 0.5])
		Wang F. et al., 2018	Emotion	Added Gaussian noise (std of 0.001~0.5)
	Recombination	Freer and Yang, 2020	Motor imagery	Segmented and recombined raw EEGs
		Cho et al., 2020		Segmented and recombined raw EEGs
		Dai et al., 2020		Segmented and recombined raw EEGs
		Huang et al., 2020		Segmented and recombined STFT images
		Fahimi et al., 2020	Motor	Segmented and recombined both raw EEGs and STFT images
		Zhao X. et al., 2020	Seizure	Segmented and recombined DCT images
		Fan et al., 2020	Sleep	Segmented and recombined raw EEGs; compared synthesizing qualities to other DA methods
		Supratak and Guo, 2020		Segmented and recombined raw EEGs
	SMOTE (Chawla et al., 2002)	Lee T. et al., 2020	ERP	Used borderline-SMOTE algorithm to raw EEGs
		Sun et al., 2019	Sleep	Used SMOTE algorithm to hand-crafted features
	Amplifying	Freer and Yang, 2020	Motor imagery	Amplified raw EEG ±2~20%
		Sakai et al., 2017	Cognition	Amplified raw EEG ±10%
	Mixup (Zhang et al., 2018b)	Kostas and Rudzicz, 2020	Multi	Conducted mixup algorithm to raw EEGs; experimented TL experiments
Intrinsic mode decomposition	EMD (Flandrin et al., 2004)	Zhang et al., 2019d	Motor imagery	Estimated and recombined IMFs of raw EEGs
		Dinarès-Ferran et al., 2018		Estimated and recombined IMFs of raw EEGs
		Kalaganis et al., 2020	Cognition	Estimated and recombined IMFs of graphs estimated by raw EEGs
	SOM (Kohonen, 1990)	Liu et al., 2016	Drowsy	Conducted ASSOM algorithm

**Table 2:** Deep generative data augmentation methods.

	**Approach**	**References**	**Paradigm**	**Summary**
GAN	GAN (Goodfellow et al., 2014)	Roy et al., 2020	Motor imagery	Devised LSTM-based generator and discriminator; qualitatively analyzed generated signals
		Krishna et al., 2020	Speech	Devised GRU-based generator and discriminator
	LSGAN (Mao et al., 2017)	Pascual et al., 2019	Seizure	Devised U-Net-based generator and discriminator; used conditional GAN concept
	DCGAN (Radford et al., 2015)	Zhang et al., 2020b	Motor imagery	Generated STFT images estimated from raw EEGs; compared synthesizing quality to other DA methods
		Zhang and Liu, 2018		Compared classification accuracy of testing dataset for different ratio of raw data and artificial data; used conditional GAN concept
		Fahimi et al., 2020	Motor	Used feature vector with the random noise for the generator input
		Lee Y. E. et al., 2020	ERP	Used features of EEG signals during walking as the generator input to reconstruct EEG signals similar to ones during standing
		Truong et al., 2019a	Seizure	Generated STFT images estimated from raw EEGs
		Truong et al., 2019b		Generated STFT images estimated from raw EEGs
		Fan et al., 2020	Sleep	Compared synthesizing quality to other DA methods
	WGAN (Arjovsky et al., 2017)	Ko et al., 2019	Motor imagery	Conducted gradient penalty rather than weight clipping; used semi-supervised GAN concept
		Hartmann et al., 2018	Motor	Conducted gradient penalty rather than weight clipping
		Aznan et al., 2019	SSVEP	Compared synthesizing quality to VAE-based DA methods; experimented TL setting
		Panwar et al., 2019b	RSVP	Conducted gradient penalty rather than weight clipping; used conditional GAN concept
		Luo et al., 2020	Emotion	Conducted gradient penalty rather than weight clipping; used conditional GAN concept
		Luo and Lu, 2018		Conducted gradient penalty rather than weight clipping; used conditional GAN concept
		Panwar et al., 2019a	Drowsy	Conducted gradient penalty rather than weight clipping
		Hwang et al., 2019	Cognition	Designed zero-calibration experiments
VAE	AE (Ballard, 1987)	Zhang et al., 2020b	Motor imagery	Generated STFT images estimated from raw EEGs; compared synthesizing quality to other DA methods
	VAE (Kingma and Welling, 2014)	Zhang et al., 2020b	Motor imagery	Generated STFT images estimated from raw EEGs; compared synthesizing quality to other DA methods
		Fahimi et al., 2020	Motor	Compared synthesizing quality to other DA methods
		Aznan et al., 2019	SSVEP	Compared synthesizing quality to VAE-based DA methods; experimented TL setting
		Luo et al., 2020	Emotion	Compared synthesizing quality to VAE-based DA methods

**Table 3:** Explicit transfer learning methods.

	**Approach**	**References**	**Paradigm**	**Summary**
Non-parametric alignment	MMD	Hang et al., 2019	Motor imagery	Minimized MMD in a feature level and introduced CDFL
		Chai et al., 2016	Emotion	Minimized MMD in a feature level and trained AE and classifier separately
	KLD	Zhang et al., 2017	Sleep	Minimized KLD in a feature level and trained with classifier in an end-to-end manner
	EA	Kostas and Rudzicz, 2020	Multi	Constrained that the mean covariance matrix becomes an identity matrix in a raw data level
Adversarial learning	A-cVAE (Wang Y. et al., 2018)	Özdenizci et al., 2019	Motor imagery	Added an adversarial network to cVAE, and trained cVAE and classifier separately
	DANN (Ganin et al., 2016)	Özdenizci et al., 2020	Motor imagery	Devised DANN by exploiting various CNN-based architectures as their feature extractor
		Zhao H. et al., 2020		Added center loss for target to minimize intra-class compactness and maximize inter-class separability
		Tang and Zhang, 2020		Fed output of a classifier into a domain discriminator
		Jeon et al., 2019		Selected source based on resting-state EEG signals
		Wei et al., 2020b	RSVP	Selected sources based on a ranking of performances in subject-specific classifiers
		Wang et al., 2021	Emotion	Selected sources based on a ranking of performances in subject-specific classifiers and devised centroid alignment loss
		Nasiri and Clifford, 2020	Sleep	Estimated attention maps using channel-wise domain discriminators
		Ma et al., 2019	Drowsy	Trained additional parameters capturing subject-specific features

**Table 4:** Implicit transfer learning methods.

	**Approach**	**References**	**Paradigm**	**Summary**
Fine-tuning	Whole	Shovon et al., 2019	Motor imagery	Pre-trained with natural images
		Aznan et al., 2019	SSVEP	Pre-trained with synthetic SSVEP samples
		Andreotti et al., 2018	Sleep	Trained their network with source subjects and fine-tuned it with target subject (LOO)
		Phan et al., 2020		Pre-trained network with different dataset
		Vilamala et al., 2017		Pre-trained network with natural images
		Fahimi et al., 2019	Cognition	Trained their network with source subjects and fine-tuned it with target subject (LOO)
	Partial	Zhang et al., 2021	Motor imagery	Fine-tuned only fully-connected layers
		Zhao et al., 2019		Conducted ablation studies to identify which layer should be transferred target
		Raghu et al., 2020	Seizure	Fine-tuned the last some layers of pre-trained network
		Olesen et al., 2020	Sleep	Fine-tuned parts of parameters
Enhancing representational power	Attention	Zhang et al., 2018a	Motor imagery	Designed a self-attention module to find more class-discriminative segments
		Zhang et al., 2019a		Designed a recurrent self-attention module
		Zhang et al., 2020a		Presented raw EEG to a spatial graph and designed a recurrent self-attention module
		Zhang et al., 2019b		Presented raw EEG to a spatial graph and designed two attention modules; one for attentive temporal dynamics and the other for attentive channels
	Multi-scale features	Kwon et al., 2019		Extracted spatio-spectral features in multi-frequency bands using CSP and selected top bands to use them as inputs
		Ko et al., 2020a	Multi	Extracted multi-scale features including spatio-temporal-spectral patterns
	Maximize mutual information	Jeon et al., 2020	Motor imagery	Decomposed an intermediate feature into a class-relevant and class-irrelevant feature and maximized mutual information between low-level and high-level representations
Meta-learning	MAML (Finn et al., 2017)	Duan et al., 2020	Multi	Trained optimal parameters through gradient-based optimization and conducted fine-tuning with a small amount of target data
	Relation (Sung et al., 2018)	An et al., 2020	Motor imagery	Estimated relation scores between support and query sets among source subjects in few-shot scenarios

## 2. Advances in Data Augmentation

### 2.1. What Is Data Augmentation?

Recently, DL-based BCIs have shown promising results in both active and passive BCI applications. However, a sufficient number of training EEG samples are required to train DL-based BCIs to avoid *overfitting* problems. DA is one way to address the data insufficiency problem. Specifically, DA increases the amount of data by synthesizing samples from the existing training data. Thus, DL models cannot overfit all samples and are forced to generalize well. Commonly, in the DL-based computer vision field (Simonyan and Zisserman, 2014; He et al., 2016), image samples are rotated/shifted/rescaled/flipped/sheared/stretched to be augmented. Further, generating extra samples from the existing ones by exploiting DL-based generative models is one of the most important strategies in DA. Because DA techniques help reduce the necessity of acquiring new EEG data, which is hindered by its cost-/time-consuming properties (Hartmann et al., 2018; Freer and Yang, 2020), they have gained significant attention in the BCI field. Here, we review the DA methodologies used for improving the performance of DL-based BCIs.

### 2.2. Challenges in Data Augmentation

A major difference between EEG data and image data is *translational invariance*, a property that an output value is invariant with respect to positional transformations of an input. Common computer vision tasks have to solve the problems of viewpoint, lightness, background, scale, etc. Therefore, in the computer vision field, widely used DA techniques, such as translation and rotation, are designed to improve the translational invariance of the training dataset. Further, those computer vision methods mostly use CNNs that exploit two-dimensional (height × width) and/or three-dimensional (height × width × depth) convolutional kernels. A CNN learns local features by sharing kernel weights, thus translational invariance is naturally followed. In other words, it represents patterns regardless of the position of the object in an input image. In contrast, for raw EEG analysis, DL-based BCIs (Schirrmeister et al., 2017; Lawhern et al., 2018; Ko et al., 2020a) are widely designed to extract features of EEG by using one-dimensional (temporal or spatial) convolution kernels. Furthermore, retraining the *spatio-spectral-temporal* information of raw EEG is also important for these DL-based BCIs. Hence, commonly used DA methods in computer vision tasks, e.g., rotating, cropping, scaling, are rarely applicable to DL-based BCIs, because those methods may harm the spectro-spatio-temporal information in EEG signals. In other words, we cannot augment raw EEG signals using simple techniques. Moreover, labeling augmented EEG samples via geometric manipulation is also difficult. In this regard, many DL methods for DL-based BCIs apply geometric manipulation to *spectrogram* images estimated from raw EEGs (Shovon et al., 2019; Zhang et al., 2020b), or cropped EEGs using a sliding window (Schirrmeister et al., 2017; Ko et al., 2018; Majidov and Whangbo, 2019). Meanwhile, other DA methods for DL-based BCIs (Hartmann et al., 2018; Luo and Lu, 2018; Hwang et al., 2019) have focused on synthesizing EEG signals from existing ones. These works generally introduce DL-based generative models (Goodfellow et al., 2014; Kingma and Welling, 2014)-based augmenting methods. However, as synthesized signals are not sufficiently realistic to be used as training samples, many studies have tried to improve the generation ability, i.e., the quality of augmented samples by regularizing their generative models (Arjovsky et al., 2017).

### 2.3. Approaches in Data Augmentation

DA methods in BCI can be categorized into two groups—geometric manipulation-based and deep generative model-based methods—depending on modifying existing samples and synthesizing novel training samples with an additional deep generative model, respectively. First, as the direct application of data modification used in computer vision to DL-based BCIs is somewhat difficult, Lotte et al. (2018) showed that geometric manipulation-based EEG DA can improve the BCI performance of linear machine learning models. Inspired by these intriguing results, in case of the geometric manipulation-based group, it was hypothesized that traditional DA techniques used in computer vision can be extended to DL-based BCIs. Further, some pioneering studies (Liu et al., 2016; Zhang et al., 2019d) have attempted to learn the intrinsic mode, i.e., subspaces of the training data, and controlled them to generate new data. Second, generative model-based approaches have gained attention from the BCI society with algorithmic advancements of generative models. DL-based generative model *explicitly*, e.g., *variational autoencoder* (VAE) (Kingma and Welling, 2014), or *implicitly*, e.g., *generative adversarial network* (GAN) (Goodfellow et al., 2014), learn the distribution of input data as well as output result. Generation of synthetic data in the input data space is possible by sampling from the learned distribution. The size of the training dataset can be considerably expanded by adopting deep generative model for BCI methods, using a limited number of samples, i.e., less than hundreds (Hartmann et al., 2018; Roy et al., 2020). In addition, some studies (Ko et al., 2019; Panwar et al., 2019a) use *min-max game*-based training algorithms, a core of GAN for DL-based BCI model training, thereby improving the BCI performance even with fewer training samples.

#### 2.3.1. Geometric Manipulation-Based Data Augmentation Methods

Geometric manipulation is one of the most simple and efficient DA ways. It modifies data without additional learning, hence is applicable directly and intuitively. Geometric manipulation-based DA methods show promising results for performance improvements in several computer vision tasks (Simonyan and Zisserman, 2014; He et al., 2016); thus, many attempts have been made to apply similar approaches to EEG data. In this section, we review many interesting DL-based BCI methods that take traditional DA strategies developed in computer vision tasks, such as geometric transformation (Schirrmeister et al., 2017), noise addition (Parvan et al., 2019), and mixup (Kostas and Rudzicz, 2020). Some studies used the segmentation and recombination approach for DA (Freer and Yang, 2020), whereas other studies learned the intrinsic modes of EEG data and generated novel samples by modifying the learned modes (Liu et al., 2016).

##### 2.3.1.1. Raw Data Modification

**Figure 2:**
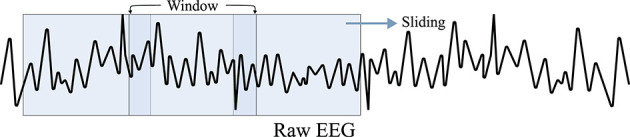
Cropping strategy using a sliding window (Schirrmeister et al., 2017; Ko et al., 2018). For a raw EEG signal, a sliding window with a length shorter than that of the signal moves on EEG with a predefined stride. Subsequently, the window crops a part of signal for augmentation.

A straightforward means of raw data modification is *geometric transformation*, which includes rotating, shifting, flipping, lightening, zooming, and cropping. As geometric transformation is easily applicable, many DL-based BCI methods use it as DA, based on Lotte et al. (2018)'s pioneering approaches, e.g., segmentation and recombination of EEG signals. For instance, Zhang et al. (2020b) performed three different geometric transformation-based DAs. First, Zhang et al. rotated spectrogram images of EEG signals estimated by using *short-time Fourier transform* (STFT). Further, they shifted the spectrogram and filled the remaining space with random noise and finally, perturbed the RGB values of the STFT image in the color space. Shovon et al. (2019) also performed DA by rotating, flipping, zooming, and brightening spectrogram images of motor imagery EEG signals. Moreover, as depicted in Figure 2, Schirrmeister et al. (2017), Ko et al. (2018), and Majidov and Whangbo (2019) used similar approaches to augment raw motor imagery EEG samples; they cropped EEG signals from an EEG epoch by using a sliding window having a shorter time length than that of the epoch. Freer and Yang (2020) performed flipping raw motor imagery samples to augment their training data. Furthermore, Mousavi et al. (2019) conducted a sliding window-based DA technique to increase the number of training EEG samples for sleep stage recognition. Supratak and Guo (2020) also focused on the sleep stage classification task but augmented the training dataset using the shifting technique. Finally, Sakai et al. (2017) used shifting to augment their cognition classification task, classifying EEG signals acquired at *motivated* status and *unmotivated* statuses.

Similar to the geometric transformation method, a *noise addition*-based DA technique has also been widely used in many successful DL-based computer vision studies (Simonyan and Zisserman, 2014; He et al., 2016). The noise addition facilitates DA by adding randomly sampled noise values to the original samples. In terms of DA for EEG, Zhang et al. (2020b) augmented spectrogram images of motor imagery EEG by adding Gaussian noise. Similarly, Parvan et al. (2019) and Freer and Yang (2020) performed noise addition using uniform distribution and Gaussian distribution to augment raw motor imagery EEG samples, respectively. Finally, Wang F. et al. (2018) added Gaussian noise to differential entropy values estimated from emotion EEG signals for the DA. Interestingly, all DL-based BCIs that exploit the noise addition method use Gaussian distribution to sample noise, with a mean value of 0 and a small standard deviation value, e.g., 0.01 or 0.001.

**Figure 3:**
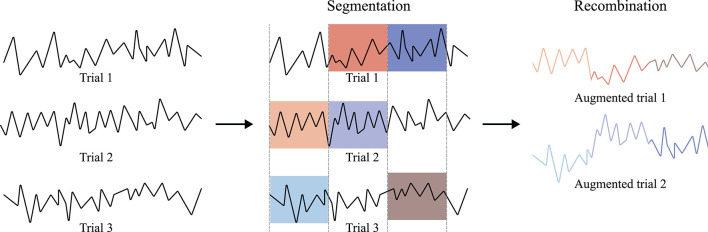
EEG segmentation and recombination method (referred to Dai et al., 2020). EEG samples are segmented into constant lengths. The divided pieces are then randomly recombined to generate new signals.

Another intuitive geometric manipulation is segmenting and recombining the EEG samples (Lotte et al., 2018). There are two methods for the segmentation and recombination methods. First, let us denote the *i*th epoch of EEG samples as **x**^*i*^. Then, with the predefined segmentation hyperparameter, *T*, the given trial is segmented to ${\text{x}}_{1}^{i},{\text{x}}_{2}^{i},...,{\text{x}}_{T}^{i}$. Finally, these segments are recombined with other segments from the other EEG epoch, i.e., **x**^*j*^, ∀*i* ≠ *j*. Thus, the augmented new sample, **x**_aug_, can be made as, for instance, ${\text{x}}_{\text{aug}}=\text{Concat}\left({\text{x}}_{1}^{1},{\text{x}}_{2}^{6},...,{\text{x}}_{T}^{4}\right)$, where Concat denotes a concatenation operation. Refer to Figure 3 for the concept of temporal signal segmentation and recombination. The other method includes spectral transformation, such as STFT. In this case, EEG samples are mapped into the spectro-temporal domain by a transformation method, segmented, and recombined. Subsequently, the augmented combinations of spectrogram segments are mapped into the temporal domain using an inverse transformation method. Recently, Cho et al. (2020), Dai et al. (2020), Freer and Yang (2020), and Huang et al. (2020) used segmentation and recombination in a temporal manner, i.e., without STFT, to augment their raw motor imagery EEG. Additionally, Huang et al. performed the same augmentation method in a spectro-temporal manner. Specifically, Huang et al. swapped entire segments in a specific frequency band of two randomly sampled EEG signals. Further, Fahimi et al. (2020) performed both segmentation and recombination methods, i.e., both temporal and spectral methods, to augment the motor execution EEG samples. Zhao X. et al. (2020) also effectively acquired artificial ictal EEG samples with a *discrete cosine transform* (DCT)-based spectral transformation. Finally, Fan et al. (2020) and Supratak and Guo (2020) performed the temporal segmentation and recombination-based DA technique to increase the training data for the sleep stage classification.

The *synthetic minority oversampling technique* (SMOTE) (Chawla et al., 2002) is one of the most widely used oversampling techniques to address the class imbalance problem in machine learning fields. Let us assume that *A* is a minority class set and its elements are **x**_*i*_ ∈ *A*. Subsequently, for each sample **x**_*i*_, we obtain its *k*-nearest neighbors, ${\text{x}}_{i}^{\left(k\right)}$, with some distance metrics, for example, Euclidean distance. Then, a new augmented sample is acquired by using ${\text{x}}_{i,\text{aug}}={\text{x}}_{i}+\epsilon |{\text{x}}_{i}-{\text{x}}_{i}^{\left(k\right)}|$ for ∀*k*, where ϵ ~ Uniform(0, 1) denotes a random number drawn from a uniform distribution. Owing to its simplicity and power, some DL-based BCI studies have used SMOTE to augment the imbalanced training data. Lee T. et al. (2020) oversampled raw *target* class EEG samples that generally belong to the minority class in the *event-related potential* (ERP) paradigm. Similarly, Romaissa et al. (2019) used SMOTE (Chawla et al., 2002) to oversample ictal EEG signals. Interestingly, Romaissa et al. first extracted the spectral features of EEG signals and performed SMOTE on the spectral domain. Sun et al. (2019) also oversampled minor epochs in the sleep stage classification by conducting SMOTE on hand-crafted features.

In addition, some studies amplified given EEG samples to augment them. Amplification-based DA can be performed by using **x**_aug_ = (1±*C*)**x**, where *C* ∈ ℝ is a predefined amplification-control hyperparameter. Freer and Yang (2020) amplified raw motor imagery samples with *C* = 0.02, 0.05, 0.1, and 0.2. Furthermore, Sakai et al. (2017) amplified EEG signals with *C* = 0.1. Sakai et al. established a 2-fold strategy of amplifying (i) all-time data and (ii) near-peak data. In the second strategy, Sakai et al. only multiplied (1±*C*) to near-peak data.

*Mixup* (Zhang et al., 2018b) is a recently proposed DA technique for computer vision tasks. For two given training samples **x**_*i*_ and **x**_*j*_, ∀*i* ≠ *j* with labels **y**_*i*_ and **y**_*j*_, respectively, an augmented sample is then estimated by using **x**_aug_ = λ**x**_*i*_ + (1 − λ)**x**_*j*_, and its label is defined as **y**_aug_ = λ**y**_*i*_ + (1 − λ)**y**_*j*_, where λ ∈ [0, 1] is a random number. In case of DL-based BCI, Kostas and Rudzicz (2020) used mixup to augment raw motor imagery/ERP/*rapid serial visual presentation* (RSVP) EEG samples and improved the BCI performance.

Raw data modification-based DA methods are easily applicable and do not require any further networks optimization. Meanwhile, because of the EEG data's spectro-spatio-temporal properties, these methods barely improve the performance and make model interpretation complicated.

##### 2.3.1.2. Intrinsic Mode Decomposition

**Figure 4:**
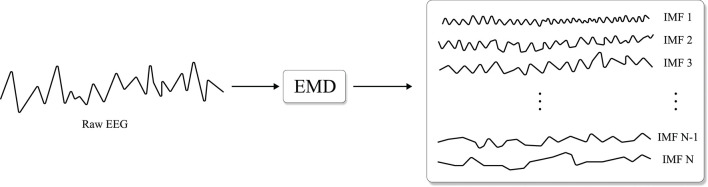
Illustration of empirical mode decomposition (EMD) (Flandrin et al., 2004) which decomposes modes, i.e., intrinsic mode function (IMF) of the input signal (re-illustrated from Dinarès-Ferran et al., 2018).

As EEG is a very non-stationary and non-linear time-series data, *empirical mode decomposition* (EMD) (Flandrin et al., 2004), illustrated in Figure 4, can be an appropriate spatio-temporal analysis method. To be specific, EEG is partitioned into *modes* called *intrinsic mode functions* (IMFs) without leaving the time domain by EMD. Similar to the segmentation and recombination, EMD-based DA first estimates IMFs of EEG signals, and IMFs are then recombined to create artificial EEG samples. Importantly, the mode of each IMF used in the DA does not overlap. Dinarès-Ferran et al. (2018) and Zhang et al. (2019d) performed EMD to acquire IMFs of motor imagery EEG samples and generated artificial samples by recombining IMFs. Kalaganis et al. (2020) created spatio-temporal graphs by using EEG signals acquired from cognitive tasks and estimated graph IMFs using EMD. Subsequently, Kalaganis et al. recombined these graph IMFs to augment the training data.

Another way to learn the intrinsic modes of the data is the *self-organizing map* (SOM) (Kohonen, 1990), which discretizes the training samples to a *map*. SOM training utilizes competitive learning. For a given training sample fed into a neural network, the Euclidean distance between each weight vector and the input data is estimated. Then, a neuron having the shortest distance is called the *best matching unit* (BMU). The weights of the BMU and neurons that are close to it in the SOM grid are adjusted to the input data. When adjusting, the magnitude of the change decreases with time and the grid-distance from the BMU. In this regard, Liu et al. (2016) applied a variant of SOM, named adaptive subspace SOM (ASSOM), trained it with predefined numbers, *N*, of quadratic modules and achieved *N* subspace representations of data **x**. Finally, *N* numbers of synthetic samples could be obtained by inversely transforming the representations. Even though intrinsic mode decomposition-based DAs effectively learn internal modes of EEG data, they still show limitations. For instance, they introduce additional hyperparameters to be found, e.g., the number of IMFs and BMUs, thus require extra tuning phase. We summarize our review of the geometric manipulation-based DA methods in Table 1.

#### 2.3.2. Generative Model-Based Data Augmentation Methods

A characteristic of generative model-based DA methods is exploiting additional DL for synthesizing training samples. Among recent successes of deep generative models, GAN (Goodfellow et al., 2014) and VAE (Kingma and Welling, 2014) demonstrate their caliber by showing practical use with sound theoretical foundations. We herein review the advances in GAN-based DA methods for BCIs (Hartmann et al., 2018; Hwang et al., 2019; Ko et al., 2019; Luo et al., 2020). These methods exploit GAN and its variants (Radford et al., 2015; Arjovsky et al., 2017; Mao et al., 2017) to learn the distribution of training samples. Those GAN-based DA methods can effectively generate artificial samples and stabilize DL-based BCI training. The autoencoder (AE) (Ballard, 1987) and VAE are also used for learning the *latent space* of the training dataset. Subsequently, some DL-based BCIs (Fahimi et al., 2020; Zhang et al., 2020b) are employed to generate artificial samples from the learned latent space, thereby augmenting the data.

##### 2.3.2.1. Generative Adversarial Network

**Figure 5:**
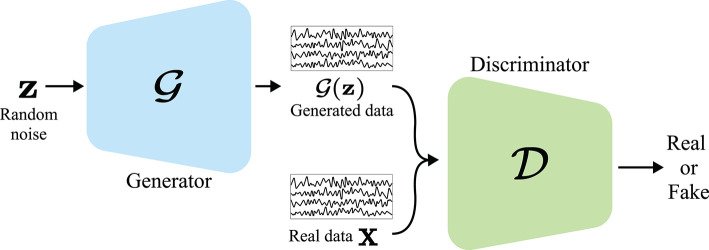
Illustration of generative adversarial network (GAN) (Goodfellow et al., 2014). Generator G outputs generated data G(z) using a random noise vector **z**. Then, discriminator D distinguishes generated data G(z) from real data **x**.

Recently, Goodfellow et al. (2014) proposed a DL-based generative model named GAN to learn deep representations of data distribution without extensively annotated training data. As depicted in Figure 5, GAN comprises two networks: a *generator* and a *discriminator*. In GAN, generator G tries to generate a *realistic* sample, G(z), from a latent code vector, **z**. Discriminator D tries to discriminate the real sample, **x**, from the generated one and outputs a probability of whether the input is real. To simultaneously train those two networks, i.e., the generator and the discriminator, GAN uses a min-max objective function:

(1) $$\begin{array}{r} \underset{D}{\max}{𝔼}_{p_{\text{x}}}\left[\log D\left(\text{x}\right)\right]+{𝔼}_{p_{\text{z}}}\left[\log\left(1-D\left(G\left(\text{z}\right)\right)\right)\right]\\ \text{and }\underset{G}{\min}{𝔼}_{p_{\text{z}}}\left[\log\left(1-D\left(G\left(\text{z}\right)\right)\right)\right], \end{array}$$

where *p*_**x**_ and *p*_**z**_ denote the distribution of real samples and latent code, respectively. In Equation (1), the Jensen-Shannon distance (JSD) is used for estimating the distance between the real sample distribution and the generated sample distribution. Here, G is minimized when D(G(z))→1, i.e., the generator tries to make realistic samples, and D is maximized when D(x)→1 and D(G(z))→0; thus, D determines the real and fake samples correctly.

Based on the use of GAN (Goodfellow et al., 2014), some DL-based BCIs use GAN as the DA method. Roy et al. (2020) proposed a GAN-based motor imagery EEG augmentation method, named *MIEEG-GAN*. Roy et al. developed an LSTM-based generator and an LSTM-based discriminator to augment both raw motor imagery EEG signals and spectrum images generated by STFT. Further, Roy et al. analyzed generated samples both qualitatively and quantitatively. Similarly, Krishna et al. (2020) constructed a *gated recurrent unit* (GRU) (Chung et al., 2014)-based generator and a GRU-based discriminator with the GAN loss function, i.e., Equation (1). Thus, Krishna et al. augmented EEG data for speech recognition and achieved performance improvement. Although these studies showed promising results for GAN-based DA, there is still room for improvement with a minor modification of the GAN loss function (Arjovsky et al., 2017); thus, many DL-based BCIs that use GAN for the DA exploited variants of GAN.

In this regard, Mao et al. (2017) proposed a modified version of the GAN loss function. They minimized the Pearson-χ^2^ distance between the real distribution and the generated data distribution instead of the JSD used for the original GAN loss function (Goodfellow et al., 2014). Thus, Mao et al. modified the loss to:

(2) $$\begin{array}{r} \underset{D}{\min}\frac{1}{2}{𝔼}_{p_{\text{x}}}\left[\log{\left(D\left(\text{x}\right)-1\right)}^{2}\right]+\frac{1}{2}{𝔼}_{p\text{z}}\left[\log{\left(D\left(G\left(\text{z}\right)\right)-0\right)}^{2}\right]\\ \text{and }\underset{G}{\min}\frac{1}{2}{𝔼}_{p_{\text{z}}}\left[\log{\left(D\left(G\left(\text{z}\right)\right)-1\right)}^{2}\right], \end{array}$$

and named their method least-squares GAN (LSGAN). This LSGAN objective function gives a larger gradient to fake samples farther from the real samples decision boundary, thereby suppressing the gradient vanishing phenomenon. In case of DA for BCI, Pascual et al. (2019) adopted LSGAN to epileptic EEG DA. Specifically, Pascual et al. used a conditional vector (Mirza and Osindero, 2014) in their model to generate ictal EEG samples from given inter-ictal EEG samples. They also exploited U-Net (Ronneberger et al., 2015) for both the generator and the discriminator. By doing so, Pascual et al. synthesized numerous ictal samples and improved the performance with the generated samples.

Meanwhile, Radford et al. (2015) focused on solving the min-max objective of GAN (Goodfellow et al., 2014) as inherently unstable. With exhaustive attempts to design a stable CNN-based GAN from scratch, Radford et al. showed that the generator of a deconvolutional network without fully-connected layers and pooling layers and the discriminator of a convolutional network without pooling layers makes GAN robust. Their successful achievement is commonly called deep convolutional GAN (DCGAN). In a BCI society, DCGAN is also widely used for DA. For instance, Zhang et al. (2020b) augmented spectrograms of motor imagery EEG estimated by applying STFT using DCGAN. Zhang and Liu (2018) also showed improved motor imagery-based BCI performance by DA using DCGAN. Fahimi et al. (2020) generated raw EEG signals using DCGAN and analyzed the generated signals using t-stochastic neighbor embedding (Maaten and Hinton, 2008) and STFT. Additionally, Lee Y. E. et al. (2020) reconstructed ERP signals using DCGAN for mobile BCI. They also showed the performance of reconstructed ERP signals and visualized the generated samples. Truong et al. (2019a,b) applied DA to STFT transforms of epileptic EEG signals using DCGAN. Finally, Fan et al. (2020) performed the DA using DCGAN to tackle a class imbalance problem in sleep staging tasks and demonstrated the validity of GAN-based DA.

Similar to LSGAN (Mao et al., 2017), Arjovsky et al. (2017) focused on changing the JSD to the Wasserstein distance. Arjovsky et al. showed that the Wasserstein distance can be applied to GAN in a theoretically rigorous manner and proposed a modified version of the objective function:

(3) $$\begin{array}{r} \underset{G}{\min}\underset{D}{\max}{𝔼}_{p_{\text{x}}}\left[D\left(\text{x}\right)\right]-{𝔼}_{p_{\text{z}}}\left[D\left(G\left(\text{z}\right)\right)\right]. \end{array}$$

To satisfy a constraint, i.e., to restrict the discriminator to the Lipschitz function, Arjovsky et al. used weight clipping on discriminator D. However, Gulrajani et al. (2017) removed the weight clipping by adding a gradient penalty regularization to the objective function and made the training stable. These methods are widely known as Wasserstein GAN (WGAN). Several researchers of DL-based BCIs showed interest in a WGAN-based DA method. Ko et al. (2019) exploited WGAN with a gradient penalty to improve the BCI performance in motor imagery. They used WGAN, rather than the DA method, for DL-based BCI model training, and improved performance even with fewer training datasets. In addition, Hartmann et al. (2018) proposed *EEG-GAN* which is a modified version of WGAN to generate artificial raw EEG data. Aznan et al. (2019) also used WGAN to augment *steady-state visual evoked potential* (SSVEP) and improved the BCI performance. Panwar et al. (2019a,b) exploited WGAN with the gradient penalty to generate raw EEG data of RSVP and drowsiness and significantly improved the BCI performance. Luo and Lu (2018) and Luo et al. (2020) modified WGAN and synthesized *differential entropy* values calculated from emotion EEG signals. As the aforementioned methods require a calibration phase, Hwang et al. (2019) tried to introduce zero-calibration. They used WGAN to generate raw EEG data acquired from a protocol of watching natural objects, such as a pizza and a banana. GAN-based DA methods synthesize realistic EEG samples by learning the data distribution implicitly, thereby showing great opportunity for DA. Nevertheless, these methods need (relatively) large amounts of data to train to network modules, i.e., the generator and the discriminator.

##### 2.3.2.2. Variational Autoencoder

**Figure 6:**
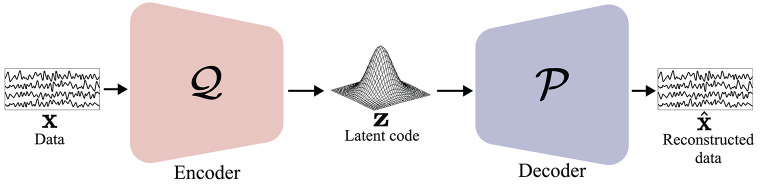
Illustration of variational autoencoder (VAE) (Kingma and Welling, 2014). Encoder Q learns latent space of input data **x**. From the learned latent space, latent code **z** is sampled and input to decoder P. Finally, decoder P reconstructs input data $\hat{\text{x}}$.

As GAN (Goodfellow et al., 2014) and its variants (Radford et al., 2015; Arjovsky et al., 2017; Mao et al., 2017) demonstrated their ability in DA, some studies focused on learning a latent representation of EEG data distribution in an explicit manner. AE (Ballard, 1987) is a neural network trained to replicate the input and the output data. AE has an encoder and a decoder; the encoder describes a *code* that is used for representing the input data, and the decoder reconstructs the input data from the code. Modern AE models have tried to generalize the encoder and the decoder functions to learn the distribution of the input data and the code. In particular, as depicted in Figure 6, VAE, which is a type of AE, learns encoder Q and decoder P through *variational inference*. The VAE (Kingma and Welling, 2014) is trained by the objective function:

(4) $$\begin{array}{r} \underset{P,Q}{\min}-{𝔼}_{Q}\left[\log\left(P\left(\text{x}|\text{z}\right)\right)\right]+\text{KLD}\left(Q\left(\text{z}|\text{x}\right)||P\left(\text{z}\right)\right). \end{array}$$

where KLD denotes the *Kullback-Leibler divergence* (KLD). In Equation (4), the first term represents a *negative log-likelihood* of the latent code, **z**, and can be considered as a reconstruction error. The second term is a regularization term to constrain the variational distribution, Q(z|x), to be similar to P(z). Based on the objective function in Equation (4), the VAE effectively represents the latent space of the data distribution and can generate novel samples from the learned latent distribution.

In this regard, some DL-based BCIs use AEs (Ballard, 1987) and VAEs (Kingma and Welling, 2014) for DA. For example, Zhang et al. (2020b) transformed EEG signals into spectrograms using STFT and reconstructed them using both an AE and a VAE. By reconstructing STFT images from the learned code, Zhang et al. could effectively acquire novel training samples. Fahimi et al. (2020) exploited a VAE to synthesize artificial motor EEG signals. Furthermore, Aznan et al. (2019) performed DA of SSVEP EEG signals using a VAE. Finally, to augment the raw emotion EEG signals, Luo et al. (2020) learned the latent space of the data distribution and generated artificial samples using a VAE. Even though VAE-based DAs learn the training data distribution and generate augmentation samples, the synthesizing quality still lacks. We summarize our review of both the GAN and VAE-based DA methods in Table 2.

## 3. Advances in Transfer Learning

### 3.1. What Is Transfer Learning?

In recent years, efforts have been made to take advantage of other real EEG samples (i.e., from a session or a subject) to train deep neural networks that decode EEG samples, thereby mitigating the data insufficiency problem (Chai et al., 2016; Andreotti et al., 2018; Fahimi et al., 2019; Özdenizci et al., 2020). These studies known as TL have focused on transferring knowledge from one dataset to another one. Generally, the TL methods aim to learn well-generalized representation among different tasks (e.g., classification, regression, clustering, etc.) or multiple datasets following different but similar distributions (i.e., domains) in other fields. Meanwhile, various TL-based BCIs have leveraged other subjects' or sessions' data to solve the same task. The representation trained from those TL methods can be applied to the seen domains (e.g., domain adaptation) or an unseen domain (e.g., domain generalization) in a short/zero-calibration manner. Hence, we mainly focus on domain adaptation/generalization-based TL approaches in this study.

### 3.2. Challenges in Transfer Learning

When designing transfer methods in BCI, there are two major concerns: (i) intra- and inter-subject variabilities and (ii) negative transfer. First, as brain signals contain their inherent background activities and vary according to their conditions, e.g., fatigue, drowsiness, excitation, and agitation, high variabilities have been observed for different subjects and even for sessions of the same subjects (Jayaram et al., 2016), which are regarded as non-stationary EEG characteristics (Chai et al., 2016; Raza and Samothrakis, 2019). In this respect, when training a DL-based BCI method with samples of one subject or session, the trained DL method cannot be deployed to another subject or session directly, because unseen data (from new subject or session) can be misaligned with the training data in the trained feature space, referred to as a *domain shift* (Ganin et al., 2016). In other words, owing to the large discrepancy between training and unseen data, the trained DL-based BCI can be degraded drastically in testing unseen data. Domain adaptation (Wang and Deng, 2018) is proposed to diminish the domain shift in other fields, such as computer vision. Owing to its goal, domain adaptation-based approaches have been widely used in DL-based BCIs (Jeon et al., 2019; Özdenizci et al., 2020; Wei et al., 2020a; Zhao H. et al., 2020). Each subject or session is regarded as one domain in most studies. Recent studies have introduced a question: what should be transferred between various domains? Although the domain-invariant features can be obtained through TL, mainly via domain adaptation techniques, it can also induce degradation of unseen data because all information is not equally transferable (Lin and Jung, 2017; Wang and Deng, 2018; Peng et al., 2019; Jeon et al., 2020), which is denoted as a *negative transfer*.

### 3.3. Approaches in Transfer Learning

TL methods in BCI can be categorized into two approaches—explicit TL and implicit TL—depending on whether to explicitly use a discrepancy between two domains in the objective function. Explicit TL-based approaches have commonly focused on minimizing a divergence between multiple domains during the training process. These methods have been fundamentally devised according to *domain theory* (Ben-David et al., 2010). In domain theory, when training a model with a labeled source domain and an unlabeled target domain, the expected error of the target domain is upper bounded as the sum of the error of the labeled source domain and the discrepancy between the source and target domains. In other words, minimizing the divergence between multiple domains is key regardless of the labels in the target domain. The question here is why TL can be considered as an effort to reduce cost/time-consuming calibration. Most studies assumed that the subject-invariant feature space can be directly applied with zero or short-calibrations for new subjects' EEG data (Jeon et al., 2020; Özdenizci et al., 2020).

Contrary to explicit TL-based methods, implicit TL-based approaches follow the hypothesis that their method can train domain-invariant feature spaces on the basis of only their internal architectures without explicitly minimizing the discrepancy. For instance, they merely perform fine-tuning with a new dataset (Andreotti et al., 2018; Fahimi et al., 2019; Zhang et al., 2021) or applied meta-learning framework (An et al., 2020; Duan et al., 2020). Furthermore, well-trained feature representation capturing multi-scale discriminative EEG patterns or focusing more discriminative temporal regions can be employed to evaluate new datasets (Kwon et al., 2019; Zhang et al., 2019a, 2020a; Ko et al., 2020a). We describe deep TL methods for zero/short-calibrations in more detail.

#### 3.3.1. Explicit Transfer Learning Methods

**Figure 7:**
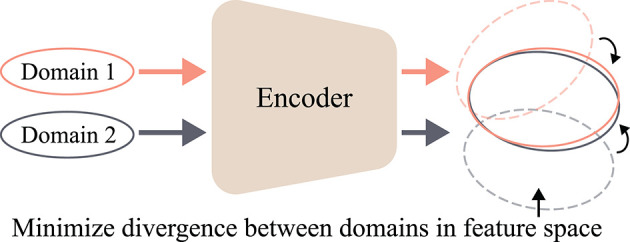
Concept of explicit TL-based methods. The alignment can be achieved by minimizing a divergence between different domains.

Explicit TL-based methods define the distributional discrepancy between multiple domains, i.e., subjects or sessions, and then minimize the discrepancy during the training by appropriately designing their objective functions, thereby achieving an alignment in the feature space. We have witnessed the success of TLs that exploit subspace alignment methods in DL-based BCIs (Chai et al., 2016; Zhang et al., 2017; Özdenizci et al., 2020; Wei et al., 2020b; Wang et al., 2021). These methods can require additional DLs (adversarial learning) or not (non-parametric). Non-parametric alignment-based methods define a distributional discrepancy between different domains at various distances (Gretton et al., 2012; He and Wu, 2019) and then minimize it during optimization. Therefore, this minimization term is considered to be a regularization on a latent feature space. In contrast, adversarial learning-based methods require at least one neural network. Subsequently, the additional network identifies the domain from which the input data is sampled and denotes it as a domain discriminator. Through the min-max game between the domain discriminator and a feature extractor, adversarial learning induces domain-invariant features (Ganin et al., 2016). The conceptual schematization of the explicit TL is shown in Figure 7.

##### 3.3.1.1. Non-parametric Alignment

To align features between different domains, three divergences are mainly introduced in DL-based BCIs: (i) *maximum mean discrepancy* (MMD) (Chai et al., 2016; Hang et al., 2019), (ii) KLD (Zhang et al., 2017), and (iii) *Euclidean distance* (Kostas and Rudzicz, 2020). First, MMD is the distance between two distributions *S* and *T* in a kernel embedding space and is defined as follows:

(5) $$\begin{array}{r} \text{MMD}\left(S,T\right)=∥\frac{1}{n_{S}}\overset{n_{S}}{\underset{i=1}{\sum }}\phi \left({\text{x}}_{i}\right)-\frac{1}{n_{T}}\overset{n_{T}}{\underset{j=1}{\sum }}\phi \left({\text{x}}_{j}\right){∥}_{H}^{2},\;{\text{x}}_{i}\sim S,\;{\text{x}}_{j}\sim T \end{array}$$

where ϕ denotes a mapping function for *reproducing kernel Hilbert space* (RKHS) and $||\cdot |{|}_{H}$ is the RKHS norm (Gretton et al., 2006). Here, *n*_*S*_ and *n*_*T*_ denote the number of samples drawn from the *S* and *T* distributions, respectively. In terms of TL for DL-based BCIs, Hang et al. utilized MMD to minimize the distance between the source and target domains in features extracted from fully-connected layers after convolutional layers. They deployed another loss function named the *center-based discriminative feature learning* (CDFL) method. CDFL is referred to as a regularization technique, that compels the distance between each sample feature and the corresponding class center point to become less than thresholds for better separability between different classes. As a result, Hang et al. acquired a domain-invariant feature of motor imagery EEG signals at the class level by minimizing MMD as well as CDFL. Chai et al. also minimized MMD in a hidden feature space among source and target samples during training an AE and obtained a domain-invariant subspace for the emotion recognition task. However, the classifier was not jointly trained with the AE.

Similar to Chai et al. (2016)'s work, Zhang et al. (2017) constrained a hidden space in their AE to train a subject-invariant feature of the sleep EEG. However, according to the existing AE-based TL method (Zhuang et al., 2015), they only reduced a symmetric KLD between the source and target features by using an identity function as ϕ in Equation (5). In other words, they did not transform their features to another space during training. Although they trained all parameters of the AE and the classifier in an end-to-end manner, their method diminished only the marginal distribution difference, disregarding the conditional distributions of the two domains in classification (Ding et al., 2018).

Kostas and Rudzicz (2020) performed raw EEG data alignment from many subjects at the preprocessing step by applying the *Euclidean alignment* (EA) method (He and Wu, 2019). As raw EEG signals can be transformed into covariance matrices, i.e., *symmetric positive definite*, they can be operated on a Riemannian manifold (Wang et al., 2021). However, He and Wu demonstrated that covariance matrix alignment on the Riemannian space for TL required high computational costs and showed unstable operations compared with the Euclidean space. For this reason, Kostas and Rudzicz constrained the mean covariance matrix to become an identity matrix according to the EA method and then used the aligned samples as the input of their TL for the DL-based BCI method. Thus, Kostas and Rudzicz developed the TL method for motor imagery, ERP, and RSVP.

These non-parametric alignment-based methods do not require additional trainable parameters whereas they can be employed between only two domains. Accordingly, they selected two subjects (i.e., source and target subject) in their dataset (Chai et al., 2016; Hang et al., 2019) or considered the remaining subjects except for a target subject as one source subject (Zhang et al., 2017; Kostas and Rudzicz, 2020). Consequently, we cannot easily utilize their methods in order for a zero-calibration BCI.

##### 3.3.1.2. Adversarial Learning

In the BCI field, many TL methods have applied an adversarial learning (Goodfellow et al., 2014) concept. Among them, the *adversarial conditional VAE* (A-cVAE) (Wang Y. et al., 2018) and *domain adversarial neural network* (DANN) (Ganin et al., 2016) have shown their potential in training domain-invariant features from cross-subjects or cross-sessions. Özdenizci et al. (2019) proposed an adversarial neural network to learn subject-invariant latent representations by using an A-cVAE. They combined a *conditional VAE* (cVAE) (Sohn et al., 2015) and an adversarial network. To be specific, in their network, an encoder and a decoder were trained to learn latent EEG representations from multiple subjects under the subjects' ID, and an adversary was trained for subject identification. These two steps are conducted alternatively so that they can learn subject-invariant EEG representations. Subsequently, the output of the frozen encoder for the same training samples was fed into a new classifier for classification. Hence, there still exists a limitation that both subject-invariant learning class-discriminative learning did not train in an end-to-end manner.

**Figure 8:**
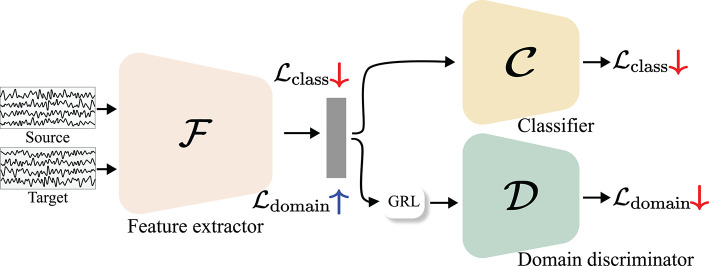
Illustration of domain adversarial neural network (DANN) (Ganin et al., 2016). $L_{\text{class}}$ and $L_{\text{domain}}$ denote a classification loss and domain loss, respectively. Through a GRL where gradients of a domain loss are reversed by multiplying a negative value, a domain loss is minimized in a domain discriminator and maximized in a feature extractor.

Most adversarial learning-based methods adopt DANN (Ganin et al., 2016) for designing their TL frameworks. DANN comprises three components a feature extractor F, domain discriminator D, and classifier C, as shown in Figure 8. The domain discriminator and the classifier identify the domains or classes to which the incoming features belong, whereas the feature extractor is trained to minimize the classification loss and maximize the domain loss through a *gradient reversal layer* (GRL) where gradients are multiplied by a negative value during the back-propagation process. The objective function of the DANN is defined as follows:

(6) $$\begin{array}{r} \underset{F,C}{\min}{𝔼}_{\text{x},\text{y}\sim p_{s}\left(\text{x},\text{y}\right)}\text{CCE}\left(C\left(F\left(\text{x}\right)\right),\text{y}\right) \end{array}$$

(7) $$\begin{array}{r} \underset{F}{\max}\underset{D}{\min}-{𝔼}_{\text{x}\sim p_{s}\left(\text{x}\right)}\left[\log D\left(F\left(\text{x}\right)\right)\right]-{𝔼}_{\text{x}\sim p_{t}\left(\text{x}\right)}\left[\log\left(1-D\left(F\left(\text{x}\right)\right)\right)\right] \end{array}$$

where **x** and **y** denote the input and corresponding labels, respectively. Here, *p*_*s*_ and *p*_*t*_ indicate distributions from the source and target domains, respectively, and CCE is the categorical cross-entropy loss that is widely used for classification tasks. Thus, Equation (6) is used to train the feature extractor F and classifier C to represent the input data and discriminate it correctly and is considered as the classification loss. In addition, in Equation (7), similar to the GAN objective function, i.e., Equation (1), feature extractor F tries to extract domain-indiscriminative features, whereas the domain discriminator D focuses on classifying the domains. In this regard, Equation (7) is commonly referred to as the domain loss. Therefore, the feature extractor output can be class-discriminative and domain-invariant by optimizing Equations (6) and (7).

Based on DANN, Özdenizci et al. (2020) introduced an adversarial learning-based TL network where the domain discriminator identifies whether features belong to which subjects, similar to the previous study of using an A-cVAE (Özdenizci et al., 2019). Özdenizci et al. demonstrated that any decoding models for EEG can be applied to their DANN-based methods by considering various CNN-based architectures (Schirrmeister et al., 2017; Lawhern et al., 2018). In this study, Özdenizci et al. effectively represented the domain-invariant features of multiple subjects' motor imagery signals.

Recently, several methods have shown that the use of only DANN (Ganin et al., 2016) has some limitations and challenges (Ma et al., 2019; Nasiri and Clifford, 2020; Tang and Zhang, 2020; Zhao H. et al., 2020). First, Zhao et al. considered a single subject as a target and the remaining subjects of datasets as source sets; therefore, the domain discriminator was trained to distinguish between the target and the sources. Furthermore, Zhao et al. exploited a classification loss and a center loss (Wen et al., 2016) for the target subject to strengthen class-discriminative power by minimizing intra-class compactness and maximizing inter-class separability. In addition, Tang and Zhang addressed that DANN cannot capture complex multimodal structures because even a perfectly trained domain discriminator cannot ensure perfect alignment between different domains. In this regard, Tang and Zhang performed an outer product between the output of the feature extractor and the output of the classifier (class probabilities) and then fed it into the domain discriminator for better alignment between the two domains according to the conditional GAN (Mirza and Osindero, 2014). Additionally, Ma et al. introduced a domain residual connection for domain generalization. They assumed that domain-invariant features and domain-specific features can be separately trained by using additional parameters in the feature extractor. In detail, the domain-invariant (denoted as common in Ma et al.'s work) parameters are shared among all source domains and the additional parameters are used only for the corresponding domain samples per domain. Subsequently, the sum between the domain-invariant outputs and the domain-specific outputs is taken as inputs of the domain discriminator and classifier. Here, the common parameters of the feature extractor and the classifier are activated on testing the unseen target's data. However, as there are no decomposition strategies, it does not ensure that the subject-specific parameters capture the real subject-specific information regardless of the subject-invariant information.

Further, to mitigate negative transfer, two approaches have been proposed: (i) source selection (Jeon et al., 2019; Wei et al., 2020b; Wang et al., 2021) and (ii) transferable attention (Nasiri and Clifford, 2020). Regarding the source selection methods, they introduced the need to obtain the most similar subjects due to the high variability between subjects. Specifically, Jeon et al. assumed that before adapting other subjects' samples, they first must select a source subject whose properties were similar to those of a target subject by performing hierarchical clustering based on resting-state EEG signal candidates in the source pool. Although their feature extractor embeds both the source and target's EEG samples to the subject-invariant representations in accordance with DANN (Ganin et al., 2016), each classifier was separately trained between source and target subjects to capture the subject-specific characteristics. Similar to Jeon et al.'s work, Wei et al. selected source subjects based on the target subject's classification performance among the source subject-specific classifiers. In detail, they first trained different classifiers for each subject and then evaluated all trained classifiers with a target subject to rank them with respect to the target subject. After ranking the performances, they selected the top *K* subjects and then used them as a source domain set. Subsequently, the classification outputs were also regarded as inputs of the domain discriminator with features in the same manner (Mirza and Osindero, 2014; Tang and Zhang, 2020). Following Wei et al.'s source selection strategy, Wang et al. trained their network with the selected sources' samples and the target samples. In Wang et al.'s work, domain adaptation was achieved by using both adversarial loss and centroid alignment loss. They considered the geometric means of each class as each class-prototype and then minimized the discrepancy between the same class-prototypes among different domains in the Riemannian space.

In the meantime, Nasiri and Clifford (2020) also described that all features can contain considerably dissimilar information among various subjects so that they are not necessarily transferable. To focus on more important or class-relevant local parts of data, Nasiri and Clifford added channel-wise domain discriminators and then used their output to generate attention maps which can be a criterion for transferability by transforming entropy.

To sum up, these adversarial learning-based methods assumed that the well-trained feature representation can be validated for unseen domains, thus, they can accomplish the zero-calibration BCI. However, in the adversarial learning-based methods, additional trainable parameters are demanded to align distributions between two or more domains. Moreover, they can cause any distortion of feature representations on account of disregarding class-related information between domains (Liu et al., 2019; Jeon et al., 2020). We summarize our review of both non-parametric alignment/adversarial learning-based TL methods in Table 3.

#### 3.3.2. Implicit Transfer Learning Methods

In this section, we describe the implicit TL approaches in DL-based BCIs. Implicit knowledge transferring methods do not explicitly minimize the discrepancy objective functions but only depend on their network (i.e., architecture). Most existing implicit TL methods have been used in the *leave-one subject-out* (LOO) scenario to fine-tune the trained parameters totally or partially using new target data (Andreotti et al., 2018; Fahimi et al., 2019; Shovon et al., 2019; Phan et al., 2020; Raghu et al., 2020; Zhang et al., 2021). Furthermore, various studies have only focused on enhancing the representational power of EEG features with only their well-designed architectures (Kwon et al., 2019; Zhang et al., 2019a; Jeon et al., 2020; Ko et al., 2020a). The remaining methods of implicit TLs (An et al., 2020; Duan et al., 2020) are based on meta-learning, which has drawn increasing attention for few-shot tasks in machine learning fields (Hospedales et al., 2020).

##### 3.3.2.1. Fine-Tuning

Fine-tuning is a retraining strategy to initialize parameters of a network as learned parameters of another identical network trained with diverse source datasets to adapt them to the target dataset. Fine-tuning can be regarded as the most naive approach to transfer knowledge. In this respect, many studies have taken advantage of fine-tuning for TL (Andreotti et al., 2018; Fahimi et al., 2019; Zhao et al., 2019; Raghu et al., 2020; Zhang et al., 2021). Deep networks have been pre-trained with multiple subjects' samples in a large source pool dataset, and entire parameters or parts of parameters have been fine-tuned to capture more target-related information. For example, Shovon et al. (2019) fine-tuned the parameters of the entire network for transferring knowledge of natural image classification tasks to motor imagery EEG classification. Specifically, they trained the pre-trained network with natural images by using STFT from motor imagery EEGs. Raghu et al. fine-tuned the last layers that were learned using the source subjects for the seizure classification task. Aznan et al. (2019) first trained a network using synthetic SSVEP samples and then fine-tuned the pre-trained network with real SSVEP samples, which leads to carrying information of synthetic SSVEP to a real SSVEP classification. In addition, Vilamala et al. (2017), Phan et al. (2020), and Andreotti et al. fine-tuned the entire network for sleep stage classification.

On the contrary to those methods, the existing works (Zhao et al., 2019; Olesen et al., 2020; Zhang et al., 2021) fine-tuned parts of the pre-trained network to transfer knowledge of EEG. For a new subject, Zhang et al. fine-tuned only the parameters of fully-connected layers while freezing the previous layers. Especially, Zhao et al. conducted ablation studies to identify which layers of their network should be transferred to the target. Whereas, those methods performed with motor imagery EEGs, Olesen et al. fine-tuned the last few layers with different samples for sleep stage classification. However, even though fine-tuning can be easily implemented, it is not performed within one process and cannot achieve zero-calibration efficiently. In addition, fine-tuning can cause over-fitting because of the small amount of target data (Kostas and Rudzicz, 2020).

##### 3.3.2.2. Enhancing Representational Power

Several studies have focused on learning better EEG representations to concentrate on more discriminative temporal slices (Zhang et al., 2018a, 2019a,b, 2020a) or capture multi-scale spatio-temporal characteristics (Kwon et al., 2019; Ko et al., 2020a) and to separate class-relevant information (Jeon et al., 2020) among diverse subjects. First, Zhang et al. investigated the temporal dynamics of EEG signals based on the attention mechanism that emphasizes on more informative region on the basis of self-relationships. In their work, raw EEG signals were first divided into various slices by applying a sliding window technique with a window size of a shorter length than the overall length of the time sequence. Next, the segmented EEG slices in the form of raw slices (Zhang et al., 2018a, 2019a) or graphs (Zhang et al., 2019b, 2020a) embedded their features via the encoding module. Subsequently, Zhang et al. used a self-attention module to obtain more class-discriminative segments among those features and then aggregated all slices by means of a weighted sum with the attention maps (Zhang et al., 2018a). Further, in order for the attentive temporal dynamics between those features, Zhang et al. (2019a, 2020a) employed a recurrent self-attention module (e.g., LSTM). Additionally, Zhang et al. (2019b) discovered more discriminative EEG channels by introducing another attention module.

Meanwhile, Kwon et al. (2019) applied band-pass filtering for various predefined frequency bands to raw EEG samples from source subjects. Subsequently, by employing a CSP (Ramoser et al., 2000), they extracted spatio-spectral features for all frequency bands. They calculated mutual information between the spatio-spectral features and class labels and then sorted mutual information of all frequency bands in the descending order. They selected the top *K* frequency bands in the list and then used them as their CNN input. Ko et al. (2020a) also demonstrated that it is of substantial importance to discover multi-scale features in terms of frequency/time ranges, considering spatial patterns. Unlike Kwon et al.'s work, Ko et al.'s network is composed of only convolutional layers; thus, it can be trained with raw EEGs in an end-to-end manner. Specifically, they first extracted spatio-temporal features in multi-scale by gathering intermediate representations of three convolutional layers and applying different spatial convolutional layers to them. After concatenating the multi-scale features, Ko et al. applied global average pooling (Lin et al., 2013) to them and fed the results to a fully-connected layer.

Jeon et al. (2020) proposed an information-theoretic method that decomposes an intermediate feature of the existing CNN models (Schirrmeister et al., 2017; Lawhern et al., 2018) into class-relevant and class-irrelevant features by estimating mutual information between them to mitigate a negative transfer. Furthermore, to enrich the representational power of their features, they maximized mutual information between class-relevant features and global features, i.e., an output of the last convolutional layer by regarding it as a more high-level representation, utilizing two mutual information neural estimators (MINEs) (Belghazi et al., 2018) from the local and global viewpoints, inspired by Hjelm et al. (2019). In detail, they exploited three MINEs (Belghazi et al., 2018); one to ensure good decomposition between class-relevant features and class-irrelevant features and the other two to make the global features contain more class-relevant information.

These methods (Kwon et al., 2019; Zhang et al., 2019a; Jeon et al., 2020; Ko et al., 2020a) have great significance in the sense that they showed the importance of exploring better EEG representation and enabled zero calibration in terms of TL. However, most of the methods for better EEG representation, except for (Ko et al., 2020a), focused on the motor imagery EEG and used the characteristics of it, which can be a limitation to apply them for other paradigms of EEG.

##### 3.3.2.3. Meta-Learning

**Figure 9:**
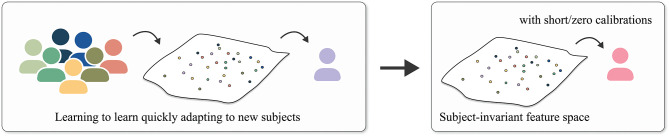
Conceptual illustration of meta-learning in BCI. Meta-learning aims to learn how to quickly adapt to new subjects by updating parameters based on a variety of tasks acquired from multiple subjects. Subsequently, the trained feature space can be considered as subject-invariant space that can be efficiently applied to new subjects with short/zero calibrations.

Meta-learning is known as *learning to learn*, which allows a model to learn a method that enables fast adaptation to a new task or environment for a few-shot learning task (Hospedales et al., 2020). After the successful application of meta-learning in machine learning fields, the meta-learning framework has recently been applied to DL-based BCIs (An et al., 2020; Duan et al., 2020). Figure 9 represents a basic concept of meta-learning with respect to TL in BCIs. As shown in Figure 9, some researchers assumed that learning to learn a task (e.g., classification, regression, etc.) among multiple subjects can result in a subject-invariant feature space that can be quickly applied to the target subject. Specifically, Duan et al. deployed a model-agnostic meta learning (MAML) (Finn et al., 2017) to obtain optimal parameters that can be rapidly adapted to target data through gradient-based optimization across multiple subjects. After dividing various source subjects' EEG data into many small groups, they updated the parameters of their network based on their gradients in two phases, meta-training and meta-test phase, and then fine-tuned the trained parameters with a small amount of target data. However, MAML easily induces over-fitting (Zintgraf et al., 2019), therefore, Duan et al. designed shallow convolutional layers for feature extraction. For this reason, their method cannot learn sufficient representation to capture class-discriminative information, which can be one of the limitations in applying their method. Another meta-learning example in BCI is the work of An et al. (2020). An et al. adopted a metric-based meta-learning framework, relation network (Sung et al., 2018), to efficiently learn class-representative features among multiple subjects. An et al. introduced three components: (i) an embedding module that extracts multi-scale features for support (labeled samples) and query (unlabeled samples) sets from source subjects, (ii) an attention module that generates a class-representative vector considering class-related importance among support sets, and (iii) a relation module to estimate the relation score between each class-representative vector and the query samples. An et al. optimized all these components by simply minimizing a cross-entropy loss, i.e., classification loss, and evaluated their network in 5-, 10-, and 20-shot settings, i.e., 5, 10, and 20 EEG samples per class. Their relational learning with attention improved the performances of all scenarios compared with a case with only relation network. However, since this metric-based meta-learning required a pair-wise input during training and evaluation, it can show difference performances depending on the support sets. We summarize our review of the fine-tuning/enhancing representational power/meta-learning-based TL methods in Table 4. Furthermore, all acronyms are listed in Appendix: List of Acronyms.

## 4. Discussion

In section 2, we review many DA methods for DL-based BCIs. From now on, we directly compare generative model-based DA methods and geometric manipulation-based DA methods and recommend a DA method for DL-based BCIs. Approximately 45% of generative model-based DA methods are reviewed, whereas ~55% of geometric manipulation-based methods are reviewed. Interestingly, Zhang et al. (2020b) and Fahimi et al. (2020) used both generative model and geometric manipulation-based DA methods. Specifically, Zhang et al. used geometric transformation, noise addition, AE (Ballard, 1987), VAE (Kingma and Welling, 2014), and DCGAN (Radford et al., 2015) to augment motor imagery data. Zhang et al. conducted classification experiments using a CNN with various real data to generate data ratio values of 1:1, 1:3, 1:5, 1:7, and 1:9. Regardless of the ratio, DCGAN-based DA achieved a high degree of consistency for the average classification accuracy whereas geometric transformation and noise addition-based methods mostly underperform with the baseline, i.e., CNN without any DA method. In addition, Fahimi et al. conducted motor execution EEG classification experiments with various augmentation methods, segmentation and recombination, VAE, and DCGAN. Similar to Zhang et al.'s work, Fahimi et al. also achieved the best performance improvement with DCGAN whereas segmentation and recombination-based augmentation did not achieve significant improvement. Based on these two results, even geometric manipulation techniques have room for improvement, and we recommend a generative model-based DA method for DL-based BCI research. Furthermore, the Wasserstein distance can be directly adapted to DCGAN, and it is expected that the BCI will have performance improvements with DCGAN trained on the Wasserstine distance (Arjovsky et al., 2017). As some pioneering studies (Hartmann et al., 2018; Hwang et al., 2019; Ko et al., 2019) have demonstrated the validity of WGAN, we anticipate that the WGAN-based DA method with careful structural design and training can improve many DL-based BCI methods.

In section 3, we summarize various TL approaches for DL-based BCIs. To achieve a short/zero calibration task, many studies performed TL across different subjects/sessions in a single dataset (Fahimi et al., 2019; Kwon et al., 2019; Özdenizci et al., 2020), inter-dataset (Phan et al., 2020), and even different data paradigms (Vilamala et al., 2017; Aznan et al., 2019). In our review, explicit TL-based methods account for nearly 45% and the remaining works are categorized as implicit TL-based methods. With regard to explicit TL-based methods, there exist two approaches, non-parametric and parametric (i.e., adversarial learning) alignment methods, for a feature space among multiple domains (subjects or sessions) (Jeon et al., 2019; Nasiri and Clifford, 2020; Özdenizci et al., 2020; Zhao H. et al., 2020; Wang et al., 2021). In Table 3, we observe that most of the existing adversarial methods employ DANN (Ganin et al., 2016). Further, modified adversarial objective functions, such as WGAN (Arjovsky et al., 2017; Gulrajani et al., 2017) and LSGAN (Mao et al., 2017), have been employed to stabilize the training process in adversarial learning-based TL approaches (Wei et al., 2020b; Zhao H. et al., 2020). In this regard, we expect that numerous variants of DANN (Tzeng et al., 2017; Xu et al., 2018; Zhang et al., 2018c; Peng et al., 2019; Wang et al., 2019) can be applied to DL-based BCI tasks. Although most implicit TL-based methods fine-tune their pre-trained network using the new target's data, there are still few limitations: (i) fine-tuning cannot reach zero-calibration and (ii) fine-tuning may lead to an overfitting problem with a small amount of target data (Kostas and Rudzicz, 2020). An et al. (2020) and Duan et al. (2020) showed successful applications of common meta-learning methods (Finn et al., 2017; Sung et al., 2018) for DL-based BCIs. However, there still remain concerns: (i) a constraint in architectures of the feature extractor (Duan et al., 2020) and (ii) variations of performances depending on varying support samples (An et al., 2020). Meanwhile, a few methods (Zhang et al., 2018a, 2019a,b, 2020a; Kwon et al., 2019; Ko et al., 2020a) demonstrated that their intrinsic architectures are sufficient to cover the new target's characteristics even in the zero-calibration scenario. Most of these methods highly rely on EEG paradigm. In this respect, despite the success of the implicit TL-based methods, there are still several points to be considered for practical applications. Hence, when first trying the short/zero-calibration BCI, we recommend the explicit TL-based methods.

Based on our survey about many pioneering DA and TL approaches for BCIs, we conclude that both strategies can be beneficial to the short- and/or zero-calibration BCIs. Especially, it can be an interesting future research direction to combine both DA and TL approaches. For instance, before performing any TL strategies, a series of DAs would augment the number of samples, thereby improving the zero-calibration BCIs. Moreover, let us assume that there exist a large amount of source data samples and a few target samples. Then, it can be considered, inter alia, some strategic TL methods to build a good starting *backbone* network. Then, DA methods are applied to the target samples to augment them. Finally, these augmented target samples can fine-tune the backbone network to improve the short-calibration BCIs.

## 5. Conclusion

In this study, we surveyed recent advances in the field of DL-based BCIs, especially for short/zero-calibration techniques. We focused on several important aspects of the short/zero-calibration techniques. Various generative model-based and geometric manipulation-based DA methods have demonstrated their promising potential in the short-calibration technique. Moreover, we summarized recent trends in TL used in DL-based BCIs. Overall, explicit TL-based and implicit TL-based TL strategies significantly improve the zero-calibration BCIs.

Presently, increasing interests in DL have considerably increased the use of BCI technologies in the *real world*. Moreover, advancements in other fields, such as computer vision will benefit from more practical and powerful DL-based BCIs. We hope that this review contributes to the BCI field as a good summary of short/zero-calibration techniques for the design of DL-based BCI studies.

## Author Contributions

WK, EJ, SJ, and JP performed the literature search. WK and EJ discussed and clustered the results and drafted the manuscript together. H-IS organized the overall structure of the manuscript and revised the manuscript, approved the final version, and agreed to be accountable for all aspects of the work. All authors contributed to the article and approved the submitted version.

## Conflict of Interest

The authors declare that the research was conducted in the absence of any commercial or financial relationships that could be construed as a potential conflict of interest.

## Figures and Tables

**Table:** 

A-cVAE	Adversarial conditional variational autoencoder
AE	Autoencoder
ASSOM	Adaptive subspace self-organizing map
BCI	Brain–computer interface
BMU	Best matching unit
BN	Batch normalization
CCE	Categorical cross-entropy
CDFL	Center-based discriminative feature learning
CNN	Convolutional neural network
CSP	Common spatial pattern
cVAE	Conditional variational autoencoder
DA	Data augmentation
DANN	Domain adversarial neural network
DCGAN	Deep convolutional generative adversarial network
DCT	Discrete cosine transform
DL	Deep learning
EA	Euclidean alignment
EEG	Electroencephalography
EMD	Empirical mode decomposition
ERP	Event-related potential
GAN	Generative adversarial network
GRL	Gradient reversal layer
GRU	Gated recurrent unit
IMF	Intrinsic mode functions
JSD	Jensen-Shannon distance
KLD	Kullback-Leibler divergence
LOO	Leave-one subject-out
LSGAN	Least square generative adversarial network
LSTM	Long-short term memory
MAML	Model-agnostic meta learning
MINE	Mutual information neural estimator
MMD	Maximum mean discrepancy
RKHS	Reproducing kernel Hilbert space
RSVP	Rapid serial visual presentation
SMOTE	Synthetic minority oversampling technique
SOM	Self-organizing map
SPD	Symmetric positive definite
SSVEP	Steady-state visual evoked potential
STFT	Short-time Fourier transform
TL	Transfer learning
VAE	Variational autoencoder
WGAN	Wasserstein generative adversarial network
